# Antioxidant White Grape Seed Phenolics: Pressurized Liquid Extracts from Different Varieties

**DOI:** 10.3390/antiox4040737

**Published:** 2015-11-19

**Authors:** Carmen Garcia-Jares, Alberto Vazquez, Juan P. Lamas, Marta Pajaro, Marta Alvarez-Casas, Marta Lores

**Affiliations:** 1 Laboratorio de Investigación y Desarrollo de Soluciones Analíticas (LIDSA), Departamento de Química Analítica, Nutrición y Bromatología, Facultad de Quimica, Universidade de Santiago de Compostela, Avda das Ciencias s/n, Campus Vida, E-15782 Santiago de Compostela, Spain; E-Mails: alberto.vazquez3@rai.usc.es (A.V.); juanpablo.lamas@usc.es (J.P.L.); martapajarovarela@gmail.com (M.P.); marta.alvarez@usc.es (M.A.-C.); 2 I-Grape Laboratory, Universidade de Santiago de Compostela, Edificio Emprendia, Campus Vida, E-15782 Santiago de Compostela, Spain

**Keywords:** grape seeds, GSE, grape marc, phenolic profile, antioxidant activity, winemaking wastes, Albariño

## Abstract

Grape seeds represent a high percentage (20% to 26%) of the grape marc obtained as a byproduct from white winemaking and keep a vast proportion of grape polyphenols. In this study, seeds obtained from 11 monovarietal white grape marcs cultivated in Northwestern Spain have been analyzed in order to characterize their polyphenolic content and antioxidant activity. Seeds of native (Albariño, Caiño, Godello, Loureiro, Torrontés, and Treixadura) and non-native (Chardonnay, Gewurtzträminer, Pinot blanc, Pinot gris, and Riesling) grape varieties have been considered. Low weight phenolics have been extracted by means of pressurized liquid extraction (PLE) and further analyzed by LC-MS/MS. The results showed that PLE extracts, whatever the grape variety of origin, contained large amounts of polyphenols and high antioxidant activity. Differences in the varietal polyphenolic profiles were found, so a selective exploitation of seeds might be possible.

## 1. Introduction

Grape seeds represent a low percentage of the fruit weight (about 5% on average), although the antioxidant phenolic compounds present in the seeds account for 60%–70% of the total polyphenols in the grape [1]. Grape marc, the main industrial byproduct from the winemaking process, is largely composed by seeds (38%–52% of dry matter) [2,3] although, frequently, they came mixed with skins and other berry residues (stalks, pulp, *etc.*) that remain after pressing the grapes.

In general, the polyphenolic content of wine depends on how the grapes are processed in the winery. The polyphenolic content of the grape marc will, therefore, also depend on the winemaking process. During red winemaking, skins and seeds are in contact with the fermenting broth for several days, conferring red wine with a high concentration of polyphenols. However, in white winemaking, the grape juice ferments without the grape marc which, thus, maintains much of its polyphenol content [4,5,6,7,8]; but it is also a byproduct with a more complex composition, making it more difficult to separate the seeds. One interesting option to isolate white grape seeds is the bio-assisted separation that occurs during the vermicomposting of grape marc on an industrial scale, yielding not only a high-quality organic vermicompost that can be used as fertilizer, but also grape seeds, which becomes a ready-to-use source of bioactive polyphenols [8,9].

The health benefits and the exploitation possibilities of plant phenolics extracts have been amply demonstrated by numerous studies over the last six decades, and are certainly based, to a large extent, on their antioxidant properties. Polyphenols from grape-derived products have been associated with the prevention of numerous diseases including cardiovascular diseases, neurodegenerative diseases, such as Alzheimer’s, as well as several forms of cancers. In particular, the grape seeds extracts (GSE) have been studied or readily used for many diverse purposes: therapy in several cardiovascular disorders [10]; reduction of oxidative stress and neuronal apoptosis related with diabetes mellitus [11]; protective activity against UVB radiation [12]; protection against early weaned stress syndrome in piglets [13]; fortification of yoghurts [14]; or even reduction of free formaldehyde at appreciable levels in the retanning process of leather [15]. There are also some studies that demonstrate the antioxidant and antibacterial activities of the GSE [16] and also the antiviral effects against a number of viruses [17]. All these varied and attractive uses have been derived in the commercialization of different GSE and, therefore, the occurrence of fundamental concerns in its quality control, already leading to the detection of adulterated products found to contain peanut skin extract [18].

The composition of grape seeds is basically (*w*/*w*) 40% fiber, 16% essential oil, 11% protein, 7% complex phenolic compounds like tannins, and other substances like sugars and minerals. There are many studies on the characterization of the polyphenolic content of GSE and most of them come to similar conclusions about their characteristic profile; independently of the extraction technique used for isolating the polyphenols from the seeds: classical procedures [19]; subcritical water in a semi-continuous mode [20]; selective enzymatic extraction [21]; ultrasound-assisted extraction (US) [22]; infrared-assisted extraction (IRAE) [23]; supercritical anti-solvent extraction (SAE) and pressurized liquid extraction (PLE) with a SPE-based clean-up step of the extract prior to injection [24]; or the analytical technique used for the characterization of grape seed extracts (GSE): HPLC-UV/VIS [23,24,25,26]; Near infrared spectroscopy (NIRS) [27,28]; LC-MS or LC-MS/MS [19,24,26] being the last one a very exhaustive study, based on QqTOF and QqQ.

In summary, the polyphenolic profile of GSE is characterized by a very important presence of flavanols and some phenolic acids (e.g., gallic, protocatechuic, and caftaric acids) although in much less extent. Flavanol monomers (catechin, epicatechin, galocatechin, and epigallocatechin) are usually the most abundant compounds, followed by procyanidins (consisting of the flavan-3-ol units catechin and epicatechin simply linked by C-C bonds or doubly linked by an additional ether bond) [10]. Gallic acid can occur as an ester at C3 of the flavan-3-ol residues giving gallocatechin-gallate (GCG) and epigallocatechin-gallate (EGC). GSE lacks of stilbenes [19]; but can have a minimum concentration of flavonols (quercetin and quercetin derivatives). However, there are not many systematic approaches to the characterization of polyphenols from seeds according to the grape variety [5,24], and in regard to white grapes, they are directly scarce [5].

The present study is framed in this context, analyzing GSEs from 11 monovarietal white grape marcs from Galicia Spanish region (native and non-native) obtained by pressurizedl extraction (PLE), and further analyzed by LC-MS/MS. Seeds of native grape varieties (Albariño, Caiño, Godello, Loureiro, Torrontés, and Treixadura) and non-native varieties (Chardonnay, Gewurtzträminer, Pinot blanc, Pinot gris, and Riesling) have been considered in the study. The ultimate goal of this work is to investigate not only the phenolic profile of the white GSE, but also their antioxidant activity in order to evaluate them as potential sources of valuable phytochemicals.

## 2. Experimental Section

### 2.1. Chemicals

Pure standards of gallic acid 99% (CAS 149-91-7), (+)catechin 99% (CAS 154-23-4), (−)epicatechin 97% (CAS 490-46-0), caftaric acid 98% (CAS 67879-58-7), (−)epicatechin-galate 98% (CAS 1257-08-5), procyanidin B1 98% (CAS 20315-25-7), procyanidin B2 98% (CAS 29106-49-8), protocatechuic acid 98% (CAS 121-33-5), caffeic acid 98% (CAS 331-39-5), quercetin 98% (CAS 117-39-5), isoquercetin (quercetin-3-glucoside) 98% (CAS 482-35-9), rutin (quercetin-3-rutinoside) 98% (CAS 153-18-4), and quercetin-3-glucuronide 98% (CAS 22688-79-5) were all supplied by Sigma-Aldrich (St. Louis, MO, USA).

Individual standard stock solutions of 2000–8000 µg·mL^−1^ were prepared in methanol. Working solutions in water containing the target analytes (1–200 µg·mL^−1^, gallic acid; 5–700 µg·mL^−1^ catechin; 25–500 µg·mL^−1^ epicatechin) were obtained by appropriate dilution. Solutions were stored at 20 °C protected from light.

Washed sea sand (200–300 µm) was supplied by Scharlau (Barcelona, Spain). Methanol HPLC grade was obtained from Panreac (Castellar del Vallès, Barcelona, Spain); acetone HPLC grade and formic acid (98%–100%) (Merck, Darmstadt, Germany) and acetonitrile (LC-MS Chromasolv, Fluka, Germany). Ultrapure water was produced in the laboratory with a Milli-Q gradient system (Millipore, Bedford, MA, USA). The Folin and Ciocalteu phenol reagent was obtained from Sigma-Aldrich (Steinheim, Germany). Other chemicals that are needed to determine the spectrophotometric indexes were DMACA (*p*-dimethylamino-cinnamaldehyde, Sigma-Aldrich (Steinheim, Germany), sodium hydroxide (NaOH, Merck), sodium nitrite (NaNO_2_, Probus, VWR International Eurolab, Llinars del Vallès, Barcelona, Spain), sodium carbonate (Na_2_CO_3_, Panreac, Castellar del Vallès, Barcelona, Spain), and aluminum trichloride (AlCl_3_, Merck). 2,2-Diphenyil-1-picrylhydrazyl (DPPH, Sigma) was used to determine the scavenging activity of the extracts.

### 2.2. Samples

Samples were kindly supplied by wineries of Galicia (Northwestern Spain) belonging to five protected DO (Monterrei, Rias Baixas, Riberia Sacra, Ribeiro, and Valdeorras), and the Experimental Station for Viticulture and Winemaking of Ribadumia (Pontevedra). Grapes or the pressed marc separated before fermentation were collected in origin (vintage years 2012 and 2013), placed into plastic freezer bags, sealed, and stored at −20 °C. Seeds were manually separated and ground in a conventional electric grinder (Moulinex) just before the analysis.

To express results in dry weight (dw), the moisture content of the samples was calculated. For that, 3 g of seeds were dried in an oven at 105 °C and weighed before and after the dryness step. This operation was carried out in triplicate.

### 2.3. Pressurized Liquid Extraction (PLE)

Extractions were performed on an ASE 150 (Dionex, Co., Sunyvale, CA, USA), equipped with 10-mL stainless steel cells and 60-mL collection vials. The procedure was previously optimized by the authors [6] and briefly consists on mixing in a mortar the ground samples with the selected dispersant (ratio 1:2), introducing the mixture into the extraction cell, where 1 g of clean sand (200–300 µm grain size) was previously placed, and filling the cell with sand. Methanol 65% was used as extracting solvent using the PLE conditions: 105 °C without preheating the cell, 1500 psi extraction pressure, flush volume of 60%, purge time 100 s, two extraction cycles, 5 min of each cycle. Extracts were diluted up to 25 mL with MeOH 65% and then passed through a 0.45 µm polyvinylidene fluoride (PVDF) filter (Simplepure, Membrane Solutions, Spring View Lane Plano, TX, USA). Two or three replicates were obtained from each seeds sample.

### 2.4. LC-MS-MS (Minus or Hyphen)

The liquid chromatographic system used was a Finnigan Surveyor™ HPLC Thermo Fisher Scientific (Madrid, Spain) equipped with a TSP AS3000 autosampler. Column was a 3.9 mm × 150 mm, 4 µm, 60 Å, Waters Nova-Pak C_18_. The injection volume was 20 µL in all cases. The mobile phase solvents were (A) 1% formic acid/water, and (B) 1% formic acid/methanol. The mobile phase gradient program started with 5% B, changed to 20% B at 20 min, and then changed to 100% B at 25 min. The entire HPLC run time was 25 min with a flow rate of 1.0 mL/min and 50 °C column temperature.

Electrospray mass spectrometry was performed with a TSQ Quantum Discovery triple-stage quadrupole mass spectrometer from Thermo Fisher Scientific. Column effluent was monitored using selected reaction monitoring (SRM). Polyphenols were detected in the negative mode using ESI (electrospray ionization) and thus, producing mainly the [M−H]^−^ pseudomolecular ions, with the exception of quercetin, quercetin-glucuronide, and quercetin-glucoside, which were detected in the positive mode. The ESI-MS/MS was operated with a scanning range of *m*/*z* 100–600. The capillary voltage was set to3.0 kV and the capillary temperature was set to 320 °C. High purity nitrogen (99.9%) was used as sheath gas and auxiliary gas at 40 psi and 10 psi and 350 °C, respectively. Argon was the collision gas at 30 psi. Identification was performed using selected reaction monitoring (SRM) in negative mode (ESI-NI) of precursor > product ion transitions. The *m*/*z* values for the parent/product ions pairs were 169/125 for gallic acid and 289/205, 289/245 for both catechin and epicatechin. Only one transition was available for gallic acid, therefore, its identity was confirmed via one transition and the retention time. The corresponding tube lens offset was 90 V and the collision energies were 20 eV for *m*/*z* 125 and 245 and 16 eV for *m*/*z* 205. Table 1 summarizes the detection conditions for each compound.

**Table 1:** LC-MS/MS analytical parameters.

Compound	Retention Time (min)	Parent Ion (*m*/*z*)	Product Ions	Collision Energy (eV)
Gallic acid	2.19	169.0 [M−H]^−^	125	26
Protocatechuic acid	3.56	152.9 [M−H]^−^	108/109	26/17
Caftaric acid	3.91	310.9 [M−H]^−^	148.9/174.9/178.9	30/19/26
Procyanidin B1	4.38	577.0 [M−H]^−^	288.9/407/424.9	26
(+)Catechin	4.88	289.0 [M−H]^−^	203.1/245	26/15
Procyanidin B2	5.38	577.0 [M−H]^−^	288.9/407.0/424.9	26
(−)Epicatechin	6.14	289.0 [M−H]^−^	203.1/245	26/15
(−)Epicatechin gallate	7.34	441.0 [M−H]^−^	125/169/289	26
Quercetin-3-glucuronide	11.14	479.0 [M+H]^+^	302.9/461.5	18/14
Quercetin-3-glucoside	11.30	465.0 [M+H]^+^	256.9/302.9	41/14
Quercetin-3-rutinoside	10.33	609.1 [M−H]^−^	178.8/270.9/300	44/56/37
Quercetin	11.47	303.1 [M+H]^+^	153.0/229.1	33/28

### 2.5. Total Polyphenols

Total polyphenols (TP) content in GSE was determined according to the Folin-Ciocalteu (FC) colorimetric method [29]. TP were quantified from a calibration curve prepared with gallic acid standard solutions in concentrations ranging from 3 to 20 mg·L^−1^ (*R*^2^ = 0.9982) and expressed as mg of gallic acid equivalents in the liquid extract (mg·L^−1^ GAE). TP sample concentrations were expressed as mg·gallic acid per g of dry weight of grape seeds (mg·gallic/g·dw).

### 2.6. DPPH Radical Scavenging Activity

2,2-Diphenyil-1-picrylhydrazyl (DPPH) scavenging activity was determined using a modified method against Trolox^®^ [30]. DPPH 0.1 mM was dissolved in 100% methanol. The GSE, 0.1 mL, were added to 3.9 mL of the methanolic DPPH solution. The mixture was shaken vigorously and allowed to stand at room temperature in the dark for 30 min. The decrease in absorbance of the resulting solution was monitored at 515 gallic at 30 min. The antiradical activity (AA) was determined using the following equation (*y* = 0.5223 *x* + 0.0276; *R*^2^ = 0.999) obtained from linear regression after plotting the A_515_ of known solutions of Trolox against concentration (0.08–1 mM). The DPPH radical scavenging activity of the PLE extracts was expressed as mM·Trolox·g^−1^ of grape seeds (dw). The radical stock solution was prepared fresh daily.

### 2.7. Statistical Analysis

Data analysis was performed using Statgraphics XV Centurion software package (Manugistics Inc, Rockville, MD, USA).

## 3. Results and Discussion

### 3.1. Polyphenolic Composition and Antioxidant Activity of Grape Seeds from Galician White Varieties

The antioxidant activity and polyphenolic content of Galician white grape seeds can be seen in Table 2, expressed as the mean value for each variety. TP values were very similar among the varietal seeds studied, ranging from 99 to 121 mg·GAE/g·dw. Similar conclusion can be obtained from the antioxidant activity, in the range 23–30 mmol·Trolox/g·dw, with Loureira and Godello seeds showing the highest AA values.

**Table 2:** Antioxidant activity and polyphenolic composition of Galician varietal white grape seed samples (mean ± standard deviation, *n* = 3–7).

	Albariño	Caiño	Godello	Loureiro	Torrontes	Treixadura
AA	23.4 ± 3.9	24.4 ± 0.4	28.0 ± 0.3	29.8 ± 2.4	24.7 ± 0.9	26.8 ± 1.8
TP	98.5 ± 11.3	99.7 ± 1.1	110 ± 10	112 ± 6	99.1 ± 1.6	121 ± 9
Gallic acid	354 ± 130	233 ± 27	140 ± 41	250 ± 111	135 ± 20	461 ± 61
Protocatechuic acid	7.5 ± 1.2	9.1 ± 1.0	8.1 ± 1.3	8.5 ± 0.8	9.1 ± 0.8	10.2 ± 1.8
Caftaric acid	15.3 ± 6.5	38.1 ± 3.5	11.9 ± 3.2	27.4 ± 3.2	11.8 ± 0.5	11.8 ± 0.5
(+)Catechin	8207 ± 1696	7424 ± 187	7091 ± 1335	6456 ± 963	2677 ± 278	8222 ± 651
(−)Epicatechin	6146 ± 1855	4085 ± 88	3402 ± 775	3049 ± 646	1994 ± 42	10,603 ± 1690
(−)Epicatechin-gallate	441 ± 95	332 ± 21	613 ± 197	322 ± 36	378 ± 67	498 ± 121
Procyanidins B1 and B3	1614 ± 272	2338 ± 164	2129 ± 409	2357 ± 327	709 ± 168	1820 ± 306
Procyanidin B2	1206 ± 163	1693 ± 42	1108 ± 211	1489 ± 401	1143 ± 61	2364 ± 398
Quercetin-3-glucoside	4.1 ± 2.1	9.3 ± 2.4	4.5 ± 1.1	5.0 ± 2.5	3.5 ± 0.6	8.8 ± 3.9
Quercetin-3-glucuronide	3.1 ± 1.3	33.3 ± 17.3	5.7 ± 1.7	9.7 ± 3.3	7.2 ± 3.2	4.7 ± 1.5
Quercetin-3-rutinoside	2.3 ± 0.2	0.9 ± 0.1	3.1 ± 0.4	1.8 ± 0.6	1.3 ± 0.1	0.4 ± 0.1
Sum of compounds	18,000 ± 2539	16,195 ± 270	14,516 ± 1623	13,976 ± 1275	7067 ± 341	24,022 ± 1884

Units: TP: mg·GAE/g·dw; AA: mmol·Trolox/g·dw; compounds: µg/g·dw.

As it was expected, flavanols were the most abundant polyphenols in the seeds extracts, representing 98%–99% of the total (quantified by chromatographic analysis). Catechin was generally at the highest concentration, followed by epicatechin and procyanidins. This composition pattern is in accordance with those previously reported for Albariño grape seeds [19], and also for Greek red varieties [31]. Catechin is quite homogeneously distributed among grape varieties, ranging from 6456 to 8221 µg/g (dw) with the exception of Torrontés seeds which show a quite lower concentration, 2677 µg/g·dw. On the contrary, epicatechin shows higher variability with the grape variety, with a mean value of 4880 µg/g (dw) and a relative standard deviation of 64%. Treixadura seeds show the highest concentration of epicatechin of about 2–5 times the other grape varieties. Epicatechin-gallate ranges from 322 to 613 µg/g (dw) with a mean value of 431 µg/g (dw), and Godello seeds with the highest content; this is in accordance with the authors previous findings on Godello grape marc [7]. Regarding procyanidins, PB1 was in general at higher concentration than PB2, with the exception of Treixadura and Torrontés grape seeds. This last variety contains much lower PB1 than the rest of the samples, 709 µg/g (dw), and also one of the lowest PB2 content. This low procyanidin content of Torrontés seeds was also found in this variety grape marcs [7].

Phenolic acids gallic, protocatechuic, and caftaric were found in low concentrations, which is in accordance with previous published data on grape seeds and peels [32]. Only gallic acid was found in the samples at concentrations higher than 100 µg/g·dw, with Treixadura and Albariño seeds containing 461 and 354 µg/g·dw, respectively.

Flavonols were present in seeds at concentrations generally below 10 µg/g·dw. Flavonol content greatly depends on the exposure of the plant to the light [33] and, thus, cannot be related to grape variety.

Regarding the sum of polyphenols determined by LC-MS/MS, it can be seen in Table 2 that Treixadura shows the highest value due to its outstanding epicatechin content, whereas Torrontés seeds show the clearly lowest polyphenolic content.

As it was previously indicated, seeds represent almost a half of the total weight of the pressed grape, which is the main industrial by-product from the winemaking process to obtain white wines [2,3]. It seemed then interesting to compare the polyphenolic content of seeds with that of the same varieties marcs. In this way, data from a previous work on the characterization of Galician grape marcs can be used for the comparison, taking into account that those data refer to a different vintage period (years 2010–2012) [7]. TP values for GSEs are about 3–4 times greater than TP content of marcs. Additionally, GSEs present a 2–4 times higher content of acids than marcs (average content of the acids sum = 120 µg/g·dw). Regarding flavanols, the average sum of compounds concentrations in marc was 3190 µg/g·dw, thus indicating that also GSEs are characterized by a higher content in these compounds (4–5 times). On the contrary, and as it was discussed, the content of flavanols in the GSEs is very low, whereas that of marc samples is about two orders of magnitude higher (1585 µg/g·dw as average). The comparison of the AA between marc and GSE indicate that seeds the AA values for seeds are about one order of magnitude higher than AA values of marcs, which reinforces the interest of seeds as a source of antioxidant compounds. The varietal differences found in grape marcs are also maintained in GSEs, with Albariño and Treixadura varieties showing the higher content in polyphenols (considered as the sum of each compound concentration), and also in the major compounds, flavanols, while Torrontés is clearly characterized by the lowest content in polyphenols in both marcs and GSEs. For the rest of the varieties, a good relationship between marc and seeds was also found.

### 3.2. Differentiation of Albariño Grape Seeds Produced in the Sub-Areas of DO Rias Baixas

Albariño is the priority variety for wine production in the Rias Baixas DO, and it is the predominant white grape variety produced in Galicia. Hence, Albariño could be considered *a priori* as the most interesting grape marc for the industrial exploitation of its polyphenolic content. Rias Baixas DO include five production subzones: Salnés, Condado do Tea, O Rosal, Ribeira do Ulla, and Soutomaior; of which Salnés accounts for the 65% of the Albariño production. The study of the polyphenolic composition of Albariño variety seeds according to the different production areas is interesting for a potential differentiated exploitation of the seeds. Albariño seeds samples have been obtained from four of the five DO Rias Baixas accounting for the majority of the production, and also from Ribeira Sacra which is a DO very different from DO Rias Baixas in terms of climate, soil, and cultivation practices.

Table 3 shows the average values of the measured parameters in each of the Albariño seeds. Samples of Albariño presented differences affecting both the AA and the polyphenolic profile, being the Ribeira Sacra seeds those showing the highest AA, TP value and polyphenols concentration expressed as sum of compounds concentrations, and O Rosal samples those showing the lowest values. Regarding flavanols, catechin, and epicatechin are the most relevant, showing a broad range of concentrations according to the origin. Ribeira Sacra seeds present the highest concentrations of about 15%–20% higher than Salnés, Condado, and Ribeira do Ulla, while O Rosal seeds present flavanol concentrations significantly lower being about 50% of the rest of seeds. Furthermore, procyanidins concentrations are greater in Ribeira Sacra seeds.

**Table 3:** Antioxidant activity and polyphenolic composition of Galician varietal Albariño grape seed samples from different origin (mean ± standard deviation, *n* = 3–4).

	Condado do Tea	O Rosal	Ribeira do Ulla	Val do Salnés	Ribeira Sacra
AA	20.8 ± 1.2	19.7 ± 0.6	25.8 ± 0.4	23.8 ± 4.4	29.3 ± 0.9
TP	95.8 ± 4.6	79.5 ± 0.5	99.1 ± 5.3	101 ± 6	117 ± 2
Gallic acid	339 ± 54	110 ± 28	393 ± 19	384 ± 82	532 ± 24
Protocatechuic acid	8.1 ± 1.1	6.7 ± 0.7	8.6 ± 0.2	7.7 ± 0.9	5.8 ± 0.6
Caftaric acid	13.6 ± 0.8	12.0 ± 1.5	12.7 ± 0.0	21.2 ± 10.8	13.1 ± 1.2
(+)Catechin	8396 ± 547	4966 ± 573	8736 ± 555	8124 ± 738	10,705 ± 51
(−)Epicatechin	5848 ± 287	2819 ± 393	6290 ± 1019	6991 ± 1750	8235 ± 108
(−)Epicatechin-gallate	375 ± 94	379 ± 32	497 ± 39	461 ± 96	543 ± 75
Procyanidins B1 and B3	1466 ± 94	1283 ± 154	1576 ± 20	1776 ± 271	1951 ± 216
Procyanidin B2	1199 ± 100	958 ± 153	1106 ± 14	1292 ± 127	1392 ± 44
Quercetin-3-glucoside	4.9 ± 1.0	1.1 ± 0.2	2.6 ± 0.0	6.1 ± 1.8	2.8 ± 0.4
Quercetin-3-glucuronide	2.8 ± 0.5	3.7 ± 2.8	3.7 ± 2.7	3.2 ± 0.9	2.6 ± 0.5
Quercetin-3-rutinoside	2.4 ± 0.3	2.0 ± 0.0	2.3 ± 0.0	2.3 ± 0.2	2.2 ± 0.1
Sum of compounds	17,655 ± 642	10,540 ± 729	18,628 ± 1161	19,068 ± 1927	23,385 ± 263

Units: TP: mg GAE/g·dw; AA: mmol Trolox/g·dw; compounds: µg/g·dw.

Regarding the acids concentrations, a similar profile can be observed for gallic acid, but it changes for the other acids. Caftaric acid is significantly higher in Salnés samples (21.2 µg/g·dw) with the other samples showing a mean of 13 µg/g·dw. Protocatechuic acid is significantly lower in Ribeira Sacra samples (5.8 µg/g·dw) respect to a mean concentration in the other samples of 7.8 µg/g·dw. Distribution of flavonols among samples is more homogeneous.

If we compare these results with those obtained by Di Lecce *et al.* [19] on Albariño samples cultivated in Catalonia (Northeastern Spain), we can conclude that the phenolic profiles are comparable, with predominance of flavanols, catechin, and epicatechin, although the concentrations these authors found in seeds were one- to five-fold lower than those obtained in the present work for Galician seeds. Procyanidins showed also similar values, thus indicating that differences could be mostly attributed to the origin and in less extent to the extraction method.

### 3.3. Polyphenolic Composition of Seeds from Foreign White Grape Varieties Cultivated in Galicia

The cultivation in Galicia of foreign grape varieties is conducted to study the adaptation and possibilities of non-native grapes to produce quality wines. Although the production is still very low, in this study we have included six varieties: Chardonnay, Gewurtzträminer, Pinot blanc, Pinot gris, Riesling, and Sauvignon blanc, that are experimentally cultivated to a limited extent at the Oenological Station in Ribadumia. Then, factors such as climate, soil, and culture techniques were the same for all the samples, so data obtained can be directly compared to obtain robust conclusions. Table 4 shows the values of AA, TP, and the individual polyphenols. Results showed that AA is very similar among varieties with only a 10% of variation. However, TP and the sum of compounds concentration show important differences among varieties. Pinot blanc and Chardonnay seeds highlight by their higher phenolic content and Riesling seeds by its lowest concentration. These differences were also found in the sum of concentrations of the individual compounds (Table 4).

**Table 4:** Antioxidant activity and polyphenolic composition of foreign experimental varietal white grape seed samples (mean ± standard deviation, *n* = 3).

	Gewürztraminer	Pinot Gris	Chardonnay	Riesling	Pinot Blanc
AA	35.8 ± 4.0	36.9 ± 0.1	38.0 ± 2.9	31.0 ± 0.4	41.3 ± 1.9
TP	133 ± 19	138 ± 3	160 ± 10	123 ± 0	168 ± 6
Gallic acid	296 ± 146	238 ± 15	829 ± 214	230 ± 31	683 ± 174
Protocatechuic acid	9.6 ± 1.5	9.7 ± 0.4	9.7 ± 1.2	11.9 ± 2.9	8.4 ± 1.5
Caftaric acid	22.2 ± 7.1	40.4 ± 0.2	20.2 ± 1.3	25.1 ± 1.8	21.5 ± 4.1
(+)Catechin	13,794 ± 2877	13,033 ± 498	12,470 ± 1000	6235 ± 74	23,091 ± 2658
(−)Epicatechin	6916 ± 1114	10,393 ± 397	19,396 ± 2867	6449 ± 483	16,043 ± 1268
(−)Epicatechin-gallate	667 ± 151	518 ± 59	626 ± 25	489 ± 107	544 ± 62
Procyanidins B1 and B3	2327 ± 580	3029 ± 294	2244 ± 335	2793 ± 425	4737 ± 207
Procyanidin B2	1264 ± 44	1973 ± 327	2508 ± 116	2467 ± 13	2744 ± 450
Quercetin-3-glucoside	4.1 ± 1.2	5.0 ± 0.2	13.4 ± 0.1	13.6 ± 3.4	3.7 ± 1.0
Quercetin-3-glucuronide	2.6 ± 0.7	2.2 ± 0.4	2.0 ± 0.1	3.3 ± 1.0	1.9 ± 0.1
Quercetin-3-rutinoside	2.6 ± 0.3	2.6 ± 0.0	1.8 ± 0.1	3.3 ± 0.3	2.5 ± 0.1
Sum of compounds	25,305 ± 3147	29,245 ± 776	38,120 ± 3065	18,722 ± 657	47,881 ± 2992

Units: TP: mg GAE/g·dw; AA: mmol Trolox/g·dw; compounds: µg/g·dw.

Flavanols were the predominant compounds in all the samples, with catechin generally at the highest concentration followed by epicatechin. This order is inversed in Chardonnay and Riesling, which is in agreement with the profiles obtained by Kammerer *et al.* in Riesling [34] and Yilmaz *et al.* in Chardonnay [35], although the concentrations were highly affected by the extraction procedure, being higher in the PLE extracts obtained in the present work. Regarding procyanidins, similar concentrations were found in all varieties, with PB1 at higher concentration than PB2, results coincident with those of Rodriguez-Montealegre *et al.* [32], who analyzed the polyphenols of skins and seeds in grapes including Riesling, Chardonnay, Sauvignon blanc, and Gewurtzträminer, among other varieties, finding PB1 at much higher concentration than PB2.

Gallic acid was found in concentrations much higher in Chardonnay and Pinot blanc, while low differences among varieties were found in protocatechuic acid. Pinot gris showed a caftaric acid concentration two times higher than its mean concentration.

Regarding flavonols, quercetin-3-glucoside showed the higher concentrations, and important differences were found among Chardonnay and Riesling (the highest concentrations) and the rest of the varieties.

A comparison between grape marcs and seeds for these varieties can be made based on previous data [7]. An average of 4566 µg/g·dw was obtained for the sum of the individual concentrations in marc samples, so seeds contain about 5–9-fold higher polyphenols than marc. Expressed as the TP value, seeds showed 3–4 times higher values. The AA for the foreign varieties GSEs was about one order of magnitude higher than in marc, which is in accordance with the relationship found for the autochthonous varietal grape marc and seeds.

## 4. Conclusions

The phenolic composition and antioxidant activity of grape seeds from 11 white grape varieties cultivated in Galicia was determined in order to their characterization. Grape seeds isolated from grape marc of native and foreign varieties all cultivated in the region were considered. Results showed varietal differences in the phenolic composition of the GSE, which would constitute a starting point for a selective exploitation of the seeds obtained from winemaking wastes.
